# Difficult Airway Management in Neonates and Infants: Knowledge of Devices and a Device-Oriented Strategy

**DOI:** 10.3389/fped.2021.654291

**Published:** 2021-05-07

**Authors:** Teiji Sawa, Atsushi Kainuma, Koichi Akiyama, Mao Kinoshita, Masayuki Shibasaki

**Affiliations:** ^1^Department of Anesthesiology, Kyoto Prefectural University of Medicine, Kyoto, Japan; ^2^Department of Anesthesia, Yodogawa Christian Hospital, Osaka, Japan

**Keywords:** difficult airway management, fiberoptic scope, infant, supraglottic airway device, video laryngoscope

## Abstract

Difficult airway management (DAM) in neonates and infants requires anesthesiologists and critical care clinicians to respond rapidly with appropriate evaluation of specific situations. Therefore, organizing information regarding DAM devices and device-oriented guidance for neonate and infant DAM treatment will help practitioners select the safest and most effective strategy. Based on DAM device information and reported literature, there are three modern options for DAM in neonates and infants that can be selected according to the anatomical difficulty and device-oriented strategy: (1) video laryngoscope (VLS), (2) supraglottic airway device (SAD), and (3) flexible fiberoptic scope (FOS). Some VLSs are equipped with small blades for infants. Advanced SADs have small sizes for infants, and some effectively function as conduits for endotracheal intubation. The smallest FOS has an outer diameter of 2.2 mm and enables intubation with endotracheal tubes with an inner diameter of 3.0 mm. DAM in neonates and infants can be improved by effectively selecting the appropriate device combination and ensuring that available providers have the necessary skills.

## Introduction

Difficult airway management (DAM) in neonates and infants requires anesthesiologists and critical care clinicians to respond rapidly because of these patients' unique physiological characteristics. Their high oxygen consumption per unit of body weight and small functional residual lung capacity are associated with an increased risk of critical hypoxia. Even if practitioners carefully provide preoxygenation, desaturation can easily occur during airway management procedures, especially in neonates and infants. Effective troubleshooting of DAM cases relies on an intimate knowledge of DAM devices and skills for practical application. Therefore, practitioners will always require ongoing education on how to effectively utilize new devices and techniques.

Three sets of pediatric difficult airway guidelines (APA1–APA3) were released by the Association of Pediatric Anesthetists of Great Britain and Ireland in 2015 (1–3): APA1 is for difficult mask ventilation during routine anesthesia, APA2 is for unanticipated difficulty in tracheal intubation during routine anesthesia induction, and APA3 is for inability to intubate and ventilate (cannot intubate/cannot ventilate [CICV]) paralyzed anesthetized patients. These guidelines are only for managing unanticipated DAM in children aged 1 to 8 years and exclude children aged <1 year. However, most potentially problematic cases of DAM in neonates and infants that practitioners encounter in clinical situations are anticipated DAM. Creating a protocol appropriate for all infant and neonate patients is challenging because anticipated difficult airways require case-by-case diagnosis of the problem and a management plan tailored to each individual. Therefore, organizing information regarding DAM devices and guidance for neonate and infant DAM will help practitioners select the safest strategies using step-by-step deduction. Based on past studies, information regarding DAM devices, and our recent experience, this review summarizes the characteristics of available DAM devices for newborns and infants and discusses strategies for DAM in these patients.

## DAM Devices for Neonates and Infants

### Video Laryngoscopes

**Figure 1:**
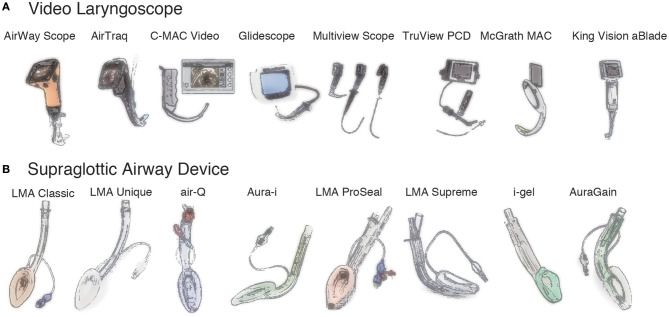
Video laryngoscopes and supraglottic airway devices. **(A)**. Video laryngoscopes. AirWay Scope® (Nihon Cohden), AirTraq™ (Prodol Meditec), C-MAC® Video (Karl Storz), Glidescope® (Verathon), Multiview Scope® (MPI), Truview PCD™ (Truphatek), McGrath MAC® (Medtronic), King Vision® aBlade® (King Systems). **(B)**. Supraglottic airway devices. LMA® Classic™ (Teleflex), LMA® Unique™ (Teleflex), air-Q® (Mercury Medical), Aura-i™ (Ambu), LMA® Proseal™ (Teleflex), LMA® Supreme™ (Teleflex), i-gel® (Intersurgical), Aura-gain™ (Ambu).

In the past, the use of VLSs was mainly limited to adults. During the last 20 years, however, pediatric VLSs have become popular (Figure 1A and Supplementary Table 1). Currently, a VLS is often the first choice for difficult intubations, even in pediatric cases. VLSs can be broadly divided into two types. The first type is equipped with an optical system in the blade with a viewing window in the body and includes the C-MAC® VLS, GlideScope®, Multiview Scope®, and Truview PCD™ Pediatric. The second type is equipped with an optical system with a viewing window and a channel for preloading an endotracheal tube in a curved blade and includes the King Vision®, AirTraq™, and AirWay Scope®.

### C-MAC® VLS

The C-MAC® VLS (Karl Storz, Tuttlingen, Germany) is one of the most popular of the first type of VLS for infant DAM because it is equipped with small metal blades for pediatric patients. Standard blades for children include Miller blade sizes #0 and #1 and BERCI-KAPLAN blade size #2. The C-MAC® D-Blade Ped is a pediatric elliptically tapered blade that curves anteriorly at the distal end for difficult intubations. The usefulness of the D-Blade has been reported for intubation of adults since 2011 (4). To date, several studies have demonstrated the advantage of the C-MAC® D-Blade for DAM in adults (5, 6). In a manikin study of normal and difficult infant airway situations, the use of two hyperangulated VLS blades, including the C-MAC® D-Blade Ped, demonstrated a shorter success time to ventilation than the use of conventional direct laryngoscopy (7). Most recently, the C-MAC® D-Blade Ped was reported to provide a better glottic view in children with simulated cervical spine injury using a manikin (8). Karl Storz recently released a single-use blade for the C-MAC® S series for pediatric use (blade sizes #0 and #1). In addition, the C-MAC® system is equipped with two types of intubation endoscope attachments (Brambrink® for an endotracheal tube [ETT] internal diameter [ID] of 2.5–3.5 mm and Bonfils® for an ETT ID of 4.0–5.5 mm). Moreover, the C-MAC® system can be equipped with a flexible fiberoptic scope (FOS) with a 2.85-mm outer diameter (OD), as described later in the FOS section of this report. Thus, the usefulness of the C-MAC® for DAM in children is expanding. Notably, however, the relatively bulky C-MAC® handle abuts the patient's chest and may prevent full insertion of the blade, as reported previously (9).

### GlideScope®

The GlideScope® (Verathon, Inc., Bothell, WA, USA) is another popular blade-type VLS. It is equipped with both reusable and single-use blades. The video monitor system (GlideScope® Core) separately equipped with a visualization system for laryngoscopy. A reusable titanium blade (GlideScope® LoPro T2 blade) is available for children weighing more than 10–15 kg. A single-use type (GlideScope® Spectrum) is available in small sizes (Miller S0, Miller S1, LoPro S1, LoPro S2, and LoPro S2.5) for neonates and infants. One study showed no difference in the intubation success rate for adult patients between the C-MAC® VLS and GlideScope® (6).

### McGrath MAC®

The McGrath MAC® (Medtronic, Dublin, Ireland) is gaining popularity. It includes a small liquid crystal display (LCD) screen and a disposable polycarbonate blade set (MAC #1, #2, #3, and #4). Blade sizes of ≥#2 are only appropriate for adults and children weighing more than 10–15 kg. The MAC® #1 blade has only recently been developed, and successful application to tracheal intubation in <3-kg infants has been reported (10).

### Multiview Scope®

The Multiview Scope® (MPI Co., Tokyo, Japan) is a unique VLS system equipped with an integrated LCD monitor and internal charge-coupled device camera with various optical intubation attachments. This device has Miller-type blade sizes #0 (MVS-ML0) and #1 (MVS-ML1) for pediatric use. The FOS attachments (MVS-FS20L and MVS-FS20S, OD of 2.1 mm) are for FOS intubation, and the three-size stylet scope attachments (MVS-SC25, OD of 2.5 mm; MVS-SC35, OD of 3.5 mm; and MVS-SC50, OD of 5.0 mm with an oxygen supply port) are for rigid optical scope intubation. There are no published studies on the Multiview Scope®, possibly because its distribution is still very limited in Japan.

### Truview PCD™ Pediatric VLS

The Truview PCD™ Pediatric VLS (Truphatek/Teleflex Medical, Morrisville, NC, USA) is equipped with metal blades (sizes #0, #1, and #2) for neonates and infants. It has a port for continuous oxygen flow that delays desaturation and prevents fogging during tracheal intubation. Several comparative studies have been performed to evaluate the Truview PCD™ Pediatric, revealing good visualization and maintenance of oxygen saturation comparable with the C-MAC® and GlideScope® (11–16).

### King Vision®

The King Vision® (King Systems, Noblesville, IN, USA) recently released the aBlade®, the lineup of which contains size #1 and #2 blades for pediatric patients. In routine use for tracheal intubation of children (≤ 2 years old), use of the aBlade® in tracheal intubation demonstrated results equivalent to those of direct laryngoscopy using a Miller blade (17).

### AirWay Scope®

The first model of the AirWay Scope® was launched by Pentax Japan (Tokyo, Japan) in 2006 (18). The present second model (AWS-S-200NK) of the AirWay Scope® VLS (Nihon Kohden Co., Tokyo, Japan) is lighter (235 g) and has a wider high-definition color LCD than the first model (285 g). In addition, it has three sizes of blades (PBLADE) for adults, children, and infants, respectively, enabling intubation using an ETT with an ID of ≧2.5 mm. The usefulness of the AirWay Scope® in pediatric difficult nasal intubation has been reported since 2010 (19, 20). A randomized controlled study suggested that the AirWay Scope® provided a similar intubation time and success rate while improving the laryngeal view compared with Macintosh laryngoscopy in children with normal airways in 2018 and 2019 (21–23).

### AirTraq™

The Rusch® AirTraq™ SP laryngoscope (Prodol Meditec S.A., Vizcaya, Spain) is equipped with size #0 and #1 blades for neonates and infants. Many comparison studies among the AirTraq™, other VLS devices, and a classic laryngoscope have been reported, and the superiority of the AirTraq™ in intubation performance has been demonstrated (24–31). The connectivity of the AirTraq™ system with other visualization systems (such as WiFi cameras, endoscopic cameras, and smartphones) was recently increased, which will probably enhance the application of the AirTraq™ in various situations.

### Head-to-Head Comparisons

Several reports have evaluated the utility of the AirTraq™ for difficult intubation of infants (32, 33). In a comparative study between AirTraq™ and GlideScope® using an infant manikin, both devices provided high-quality views of the glottis and facilitated successful tracheal intubation (31). One report indicated that use of the Airway Scope® resulted in shorter times to view the glottis and more frequent successful tracheal intubation compared with the AirTraq™ (34). When the AirWay Scope®, AirTraq™, and Miller laryngoscope were compared for tracheal intubation by novice doctors with and without simulated infant cardiopulmonary resuscitation, only the Airway Scope® was associated with successful intubation by all participants during chest compression with no substantial lengthening of the intubation time compared with the no-compression condition (35). Another study was performed to evaluate the efficacy of the AirWay Scope® for training in pediatric intubation, and the authors recommended the inclusion of both direct laryngoscopy and the AirWay Scope® in pediatric residency programs for safer and more reliable intubation (20). Fujiwara et al. (36) performed a randomized crossover trial comparing the AirWay Scope® with the GlideScope® for infant tracheal intubation by anesthesiologists during cardiopulmonary arrest simulation. They concluded that the AirWay Scope® performed better than the GlideScope® for endotracheal intubation with chest compression (36). A limitation of all these VLSs is that they require a sufficiently large mouth opening and oral space to insert the blade. Therefore, trismus and masses in the oral cavity are obstacles to intubation using a VLS. As Wallace and Engelhardt (37) summarized in their review, each VLS has its proposed benefits, but all come with potential drawbacks, and there is not a single type of VLS that suits all children or airway challenges.

## Supraglottic Airway Devices

Many different types of SADs, including reusable and single-use versions, have recently become available, and most include pediatric sizes (Figure 1B and Supplementary Table 2). In general, size #1 is for neonates and infants weighing <5 kg, while size #1.5 is for larger infants weighing 5 to 10 kg. SADs can be classified as first- or second-generation devices; the latter is equipped with a gastric drainage port. Another important characteristic of SADs is whether the device can be used as a conduit for tracheal intubation.

### Laryngeal Mask Airway® (LMA®)

The LMA® (Teleflex Medical) is the original SAD and is currently available in various designs such as the LMA® Classic™, LMA® Unique™, LMA® Proseal™, and LMA® Supreme™. Each of these is available in small sizes (sizes #1, #1.5, and #2) for neonates and infants. The LMA® Fastrach™, which was introduced in 1998, is a conduit for tracheal intubation of difficult airways but is available only in sizes larger than #3 for adult use.

### air-Q®

The air-Q® (Mercury Medical, Clearwater, FL, USA) was introduced in 2009 and can be used as a conduit for FOS intubation in children (38), infants (39), and neonates (40) with anticipated difficult airways. A meta-analysis on use of the air-Q® for guidance of intubation in pediatric patients demonstrated that the air-Q® could provide a better fiberoptic bronchoscopic view (41). Even in blind tracheal intubation, the air-Q® was shown to be a good alternative for fiberoptic-guided intubation (42). It is now available in both disposable and reusable versions. In addition to a standard cuff option, the manufacturer recently released a self-pressurizing cuff version, the air-Q®sp. The air-Q® has small sizes (#1.0, #1.5, and #2.0) for infants and children. This device is designed to enhance successful endotracheal intubation through the airway tube in combination with disposable or reusable removal stylets (for sizes above #1.0). For example, a removable connector enhances direct access to the airway tube, and the elevation ramp at the outlet of the tube directs the ETT toward the laryngeal inlet. The air-Q® size #0.5 was recently released and successfully used in a low-birth-weight neonate (43). Both retrospective and prospective studies have demonstrated acceptable clinical performance in infants and children with spontaneous and positive-pressure ventilation (44, 45).

### Ambu® Aura-i™ and Aura-gain™

The Ambu® Aura-i™ (Ambu, Copenhagen, Denmark) has an anatomical curve ensuring easy and rapid placement with intubation capability using standard ETTs. The Ambu® Aura-gain™ has an integrated gastric access port with intubation capability using standard ETTs.

### i-gel®

The i-gel® (Intersurgical, Berkshire, UK) is made from a thermoplastic elastomer designed to create a non-inflatable anatomical seal of the pharyngeal, laryngeal, and perilaryngeal structures while avoiding compression trauma. It is equipped with a gastric port and functions as a conduit for tracheal intubation.

### Head-to-Head Comparisons

A series of randomized studies compared the air-Q® intubating laryngeal airway with the LMA® Unique™ (46), Ambu® Aura-i™ (47, 48), and i-gel® (49) in healthy children scheduled for elective surgery. Comparisons between the i-gel® and other SADs have also been reported (50–57). In one study, although there was no statistically significant difference in the ease of device insertion, time to ventilation, gastric insufflation, or ventilation parameters between the air-Q® and the LMA® Unique™, the air-Q® had higher airway leak pressures and superior fiberoptic grades of view (58). Both the air-Q® and Aura-i™ devices served as effective conduits for FOS-guided tracheal intubation (46, 47). Similarly, both the air-Q® and i-gel® supraglottic airways served as effective conduits for FOS-guided tracheal intubation in children when performed by trainees with limited experience. However, the i-gel® was associated with more complications during device removal following tracheal intubation (49). Many studies have shown the usefulness of the air-Q® for both controlled ventilation and FOS-guided intubation in infants and small children <2 years old (45, 59–62).

## FOS

FOSs used for tracheal intubation can be classified into two categories: rigid and flexible (Supplementary Table 3).

### Intubation Endoscopes

Karl Storz manufactures two types of rigid FOSs: the Brambrink® and the Bonfils® intubation endoscopes. The OD of the former is 2.0 mm, which allows tracheal intubation using ETTs with an ID of 2.5 to 3.5 mm. The OD of the Bonfils® scope is 3.5 mm, enabling tracheal intubation using ETTs with an ID of 4.0 to 5.5 mm. Bein et al. (63) evaluated the pediatric Bonfils® FOS for elective endotracheal intubation in 54 children. They found an overall first-attempt success rate of 74% as well as extended intubation times (median of approximately 60 s), suggesting significant drawbacks when used for intubation of normal pediatric airways. However, a randomized controlled comparison among direct laryngoscopy, the Bonfils® FOS, and the GlideScope® Cobalt AVL VLS for visualization of the larynx and intubation of the trachea in infants and small children with normal airways concluded that the Bonfils® FOS significantly improved the view of the larynx compared with both alternatives and enabled a shorter intubation time than the GlideScope® (64, 65).

### Flexible FOS

**Figure 2:**
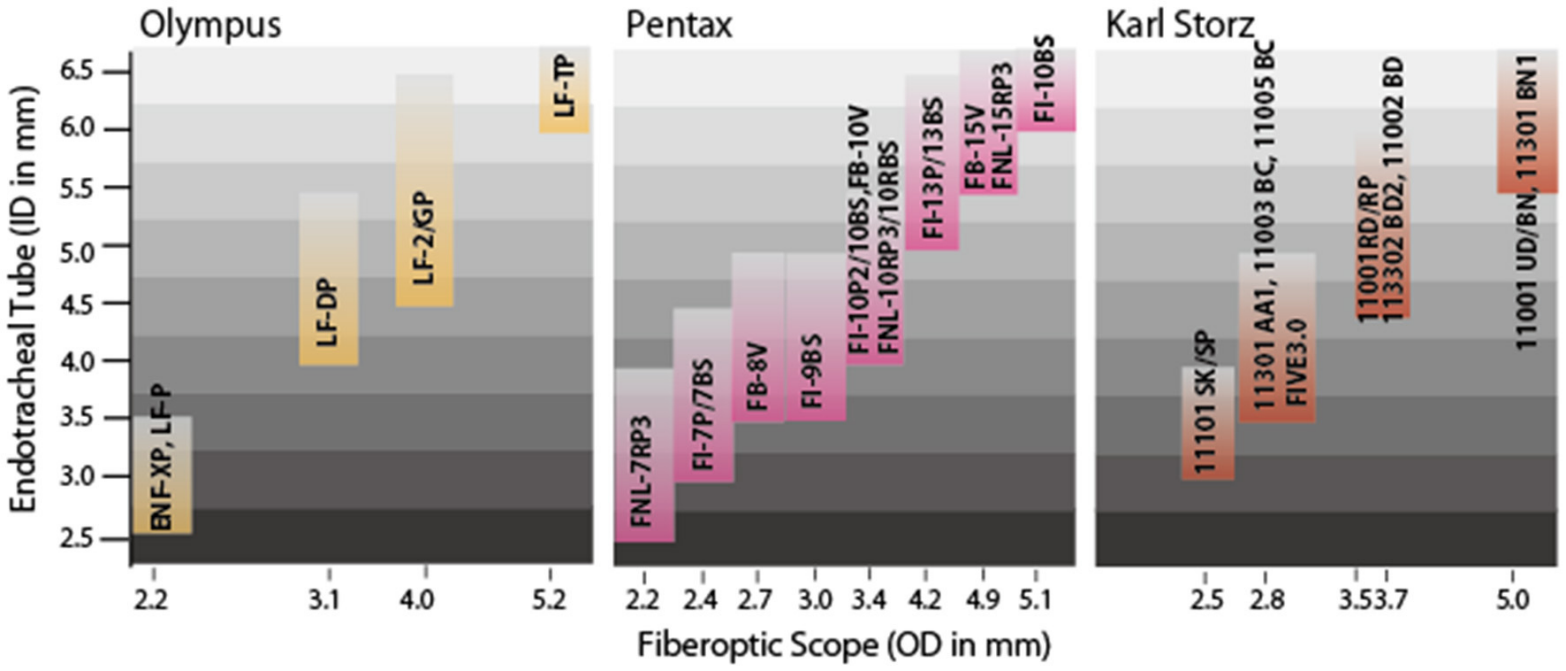
Sizes of endotracheal tubes [inner diameter (ID) in mm] and compatible fiberoptic scopes (FOSs) [outer diameter (OD) in mm]. Three major companies produce FOSs thin enough for difficult airway management of neonates and infants. The three smallest FOSs (Olympus ENF-XP® and LF-P®; Pentax FNL-7RP3®) all have an OD of 2.2 mm, enabling FOS-guided endotracheal intubation using an endotracheal tube with an ID of 3.0 mm. Note that the lower limit of the tracheal tube size is defined by the OD of the FOS, while the upper limit of the tracheal tube size is about 1 to 2 mm larger than the OD of the FOS. To ensure smooth, successful intubation, however, the ID of the endotracheal tube should ideally be 0.5 to 1.0 mm larger than the OD of the FOS.

Olympus (Tokyo, Japan), Pentax, and Karl Storz provide various flexible FOSs that can be used to intubate pediatric patients (Supplementary Table 3 and Figure 2). The smallest ones from Olympus (ENF-XP® and LF-P®) and Pentax (FNL-7RP3) have an OD of 2.2 mm, enabling tracheal intubation of ETTs with an ID of 3.0 mm. The Storz FIVE 3.0 of the C-MAC® system has an OD of 2.85 mm and allows the performance of fiberoptic intubation using an ETT with an ID of 3.0 mm (compatible with limited manufacturers). Notably, the ID of the ETT should ideally be 0.5 to 1.0 mm larger than the OD of the FOS. In 1995, Wrigley et al. (66) reported a technique using the Olympus LF-P® as an aid to oral tracheal intubation in anesthetized, spontaneously breathing children (ages 6 months to 7 years). The technique was successful in 30 of 40 patients (75%), and complications such as laryngospasm and dislocation of the fiberscope from the trachea occurred in the remaining 10 patients (25%) (66). This report suggests that the success rate and complications of FOS-guided tracheal intubation must be considered in clinical situations.

## Device-oriented Strategy for DAM in Infants

Aida et al. (67) recently performed a retrospective analysis of the incidence of difficult intubation and airway management in infants undergoing general anesthesia. Among 753 procedures in 513 infants, Cormack–Lehane grade 3 and 4 were seen in 1.2% of cases, and difficult intubation occurred in 2.4% of cases (67). The authors concluded that although muscle relaxants are useful for facilitating tracheal intubation, careful preparation of other airway devices is required for infants with predicted difficult intubation. Regarding infantile DAM, we recently treated two cases, each requiring a unique strategy according to specific anatomic challenges (68, 69). In these cases, the combination of decisions regarding sedation management (awake, light sedation, or intravenous general anesthesia), use of a SAD, or use of a FOS helped overcome these anatomic challenges. In these cases, prior to the initiation of the airway management procedure, we needed to decide whether to perform fully awake intubation, use a sedative including inhalation of volatile anesthetics, or introduce general anesthesia. This decision was linked to the judgment of whether assisted ventilation can be performed while keeping spontaneous breathing or whether positive pressure ventilation after the disappearance of spontaneous breathing is safely possible. The topical use of a local anesthetic to the larynx and vocal cords should also be considered in awake or semi-awake intubation. Muscle relaxants can make intubation easier in patients with altered anatomy or airway stenosis; however, their use can create a condition in which oxygenation cannot be ensured if spontaneous breathing is lost. Therefore, in DAM cases, we should consider rapidly reversible agent rocuronium in combination with the preparation of reversal agent sugammadex when using a muscular relaxant.

In this report, we have discussed DAM strategies for newborns and infants, focusing on three main devices (VLS, SAD, and FOB) with reference to past reports. Recent improvements in VLSs and FOBs have involved the application of advanced charge-coupled device image sensors and the evolution of small color display technology to medical devices. In addition, the adaptation of SADs for use with these video devices has evolved in recent years, further improving outcomes in cases of DAM. In addition to making decisions regarding these three tools, the clinician still has various points to consider and technologies to choose from in cases of DAM. For example, high-frequency jet ventilation might be used to improve oxygenation and ventilation. In addition, extracorporeal life support, which can help in securing the airway in exceptional conditions like long segment tracheal stenoses, may be effective with cooperation of a pediatric cardiac surgeon and a perfusionist; in 2016, we adapted cardiopulmonary bypass for infant DAM (70).

**Figure 3:**
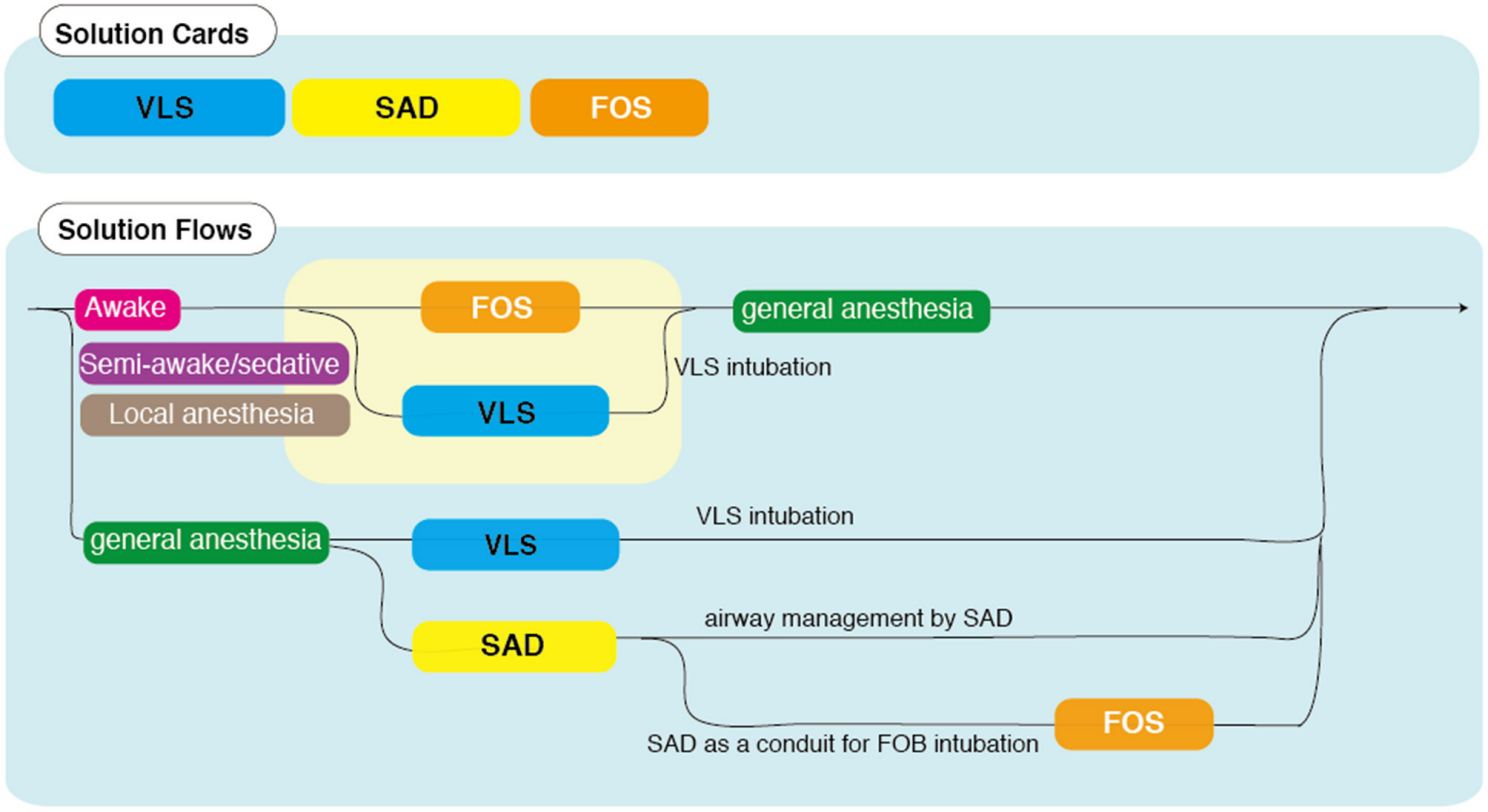
Flow chart of difficult airway management (DAM) in infants. This flow chart guides optimal treatment using different DAM devices [video laryngoscopes (VLSs), supraglottic airway devices (SADs), and fiberoptic scopes (FOSs)] in combination with the choice of consciousness management (awake, semi-awake, sedative/local anesthesia, or general anesthesia). Karl Storz FIVE 3.0 suitable for ID of 3.0 mm (compatible with limited manufacturers) and above.

Aside from exceptional measures such as the adaptation of conventional technology such as high-frequency jet ventilation and more invasive cardiopulmonary bypass, there are three modern options for DAM in neonates and infants that can be selected according to the anatomical difficulty and device-oriented strategy: the VLS, SAD, and FOS (Supplementary Table 4). The VLS can facilitate intubation in most cases with anticipated secure mask ventilation, while the SAD and FOS can help manage cases with no guarantee of safe mask ventilation. While attempting to secure the airway, patients with anatomical anomalies above the larynx can be managed with a SAD, FOS, or a combination of the two. Intubation can be obtained with FOS with or without the aid of a SAD. Another option is to perform a tracheostomy while ensuring ventilation and oxygenation with the SAD. A simple flow chart is presented in Figure 3. Decision-making must start with the choice of sedation management (awake, semi-awake, sedative/local anesthesia, or general anesthesia). Note that the flow chart shows only typical cases and the corresponding solutions as a reference. However, a thorough understanding of device characteristics and appropriate preparation is required to ensure successful outcomes of DAM.

## Data Availability Statement

The original contributions generated for the study are included in the article/Supplementary Material, further inquiries can be directed to the corresponding author/s.

## Author Contributions

TS performed the data collection, data analysis, and literature search and drafted the manuscript. AK, KA, MK, and MS prepared the manuscript and approved the final version of the manuscript to be published. All authors contributed to the article and approved the submitted version.

## Conflict of Interest

The authors declare that the research was conducted in the absence of any commercial or financial relationships that could be construed as a potential conflict of interest.
