# Supplementary material for: Difficult Airway Management in Neonates and Infants: Knowledge of Devices and a Device-Oriented Strategy
Source: Front Pediatr. 2021 May 7;9:654291. doi: 10.3389/fped.2021.654291 (PMC8138561; doi:10.3389/fped.2021.654291)
Supplement: Supplementary file 1 [file Data_Sheet_1.PDF]

Supplementary Table S1. Video laryngoscopes for difficult airway management of neonates and infants

Item	Company	Set-up for infants	Endotracheal tube size
C-MAC® Video Laryngoscope<br><br>C-MAC® S Pediatric IMAGER	Karl Storz GmbH & Co.	**Miller #0 (8401 GXC)**<br>**Miller #1 (8401 DXC)**<br>BERCI-KAPLAN #2 (88401 KXC)<br>D-Blade PED (8401 HXP)<br>**disposable Miller #0**<br>**disposable Miller #1**	-
Glidescope AVL®<br>(Reuse, & Single use)	Verathon, Inc	Reuse: GVL 2<br>**Single use::GVL 0, 1**, 2	-
McGrath MAC®	Covidien	MAC **1**, 2, 3, 4 blade	-
Multiview Scope®	MPI Co.	Miller-type size #0 (MVS-ML0)<br>Miller-type size #1 (MVS-ML1)	-
TruView PCD Pediatric®	Truphatek/Teleflex Medical	**Size #0 set** (4163E3S) for 0.8–4 kg<br>**Size #1 set** (4168E3S) for 4–8 kg<br>Size #2 set (4165E3S) Size #3 set (4161E3S) for 8–60 kg	-
King Vision aBlade®	King Systems	**#1 (KVLAB1)** for ≦ 3 yo<br>#2(KVLAB2) for 1-10 yo<br>#2C (KVLAB2C) channeled	-
AirWay Scope®<br>(AWS-S100L,<br>AWS S-200NK)	Nihon Kohden, Co.	**PBLADE (ITL-NL NEONATE)**	Below ID 5.0 mm (Without cuff)
		PBLADE (M-ITL-PL PEDIATRIC)	ID 5.5–7.6 mm (Without cuff)
AirTraq®	Prodol Meditec	**Infant Size 0**<br>A-041/ATQ-041	ID 2.5–3.5 mm
		**Pediatric Size 1**<br>A-031/ATQ-031	ID 4.0–5.5 mm

Bold text: available in neonate and infant sizes. ID, inner diameter
