# Supplementary material for: Difficult Airway Management in Neonates and Infants: Knowledge of Devices and a Device-Oriented Strategy
Source: Front Pediatr. 2021 May 7;9:654291. doi: 10.3389/fped.2021.654291 (PMC8138561; doi:10.3389/fped.2021.654291)
Supplement: Supplementary file 2 [file Data_Sheet_2.PDF]

Supplementary Table S2. Supraglottic airway devices for difficult airway management of neonates and infants

	Device	Company	Re-use	Size	Patient size	Gastric access channel	Endotracheal tube size (Conduit for tracheal intubation)
First generation	LMA Classic®	Teleflex	+	**#1**<br>**#1.5**<br>#2	**Neonates/infants up to 5 kg**<br>**Infants 5–10 kg**<br>Infants/children 10–20 kg	–	–
	LMA Unique®		–	**#1**<br>**#1.5**<br>#2	**Neonates/infants up to 5 kg**<br>**Infants 5–10 kg**<br>Infants/children 10–20 kg	–	–
	air-Q®, air-Q sp®	Mercury Medical	–	**#0.5**<br>**#1**<br>**#1.5**	**(sp) Infants <4 kg**<br>**Infants/children <7 kg**<br>Infants/children 7–17 kg	–	ID 3.0 mm uncuffed<br>ID 3.5 mm uncuffed<br>ID 4.0 mm uncuffed
	Aura-i®	Ambu	–	**#1**<br>**#1.5**<br>#2	**Infants <5 kg**<br>**Infants 5–10 kg**<br>Infants/children 10–20 kg	–	ID 3.5 mm uncuffed<br>ID 4.0 mm uncuffed<br>ID 5.0 mm uncuffed
Second generation	LMA ProSeal®	Teleflex	+	**#1**<br>**#1.5**<br>#2	**Neonates/infants up to 5 kg**<br>**Infants 5–10 kg**<br>Infants/children 10–20 kg	+	–
	LMA Supreme®		–	**#1**<br>**#1.5**<br>#2	**Neonates/infants up to 5 kg**<br>**Infants 5–10 kg**<br>Infants/children 10–20 kg	+	–
	i-gel®	Intersurgical	–	**#1**<br>**#1.5**<br>#2	**Infants 2–5 kg**<br>**Infants/children 5–12 kg**<br>Infants/children 10−25 kg	–<br>+<br>+	ID 3.0 mm<br>ID 4.0 mm<br>ID 5.0 mm
	AuraGain®	Ambu	–	**#1**<br>**#1.5**<br>#2	**Infants <5 kg**<br>**Infants/children 5–10 kg**<br>Infants/children 10–20 kg	+	ID 3.5 mm uncuffed<br>ID 4.0 mm uncuffed<br>ID 5.0 mm uncuffed

Bold text: available in neonate and infant sizes. ID, inner diameter
