# Supplementary material for: Difficult Airway Management in Neonates and Infants: Knowledge of Devices and a Device-Oriented Strategy
Source: Front Pediatr. 2021 May 7;9:654291. doi: 10.3389/fped.2021.654291 (PMC8138561; doi:10.3389/fped.2021.654291)
Supplement: Supplementary file 3 [file Data_Sheet_3.PDF]

Supplementary Table S3. Commercially available fiberoptic scopes and intubation endoscopes

Type	OD (mm)	Vendor	Name	Product type	Working length (cm)	Channel (Port)	Tip deflection up/down
rigid FOS	2.0	Karl Storz	BRAMBRINK® Intubation Endoscope	11605 C & 11605 CV (ETT ID 2.5–3.5 mm)	22	-	distal bending 40°, angle of view 100°
	3.5		BONFILS® Intubation Endoscope	10332 B & 10332 BD (ETT ID 4.0–5.5 mm)	35	-	distal bending 40°
	2.5<br>3.5<br>5.0	MPI Co.	Multiview Scope®, Stylet scope attachment	MVS-SC25 (OD 2.5 mm)<br>MVS-SC35 (OD 3.5 mm)<br>MVS-SC50 (OD 5.0 mm)	36<br>38.5<br>40	oxygen supply port	-
	2.1		Multiview Scope®, Fiberscope attachment	MVS-FS20L<br>MVS-FS20S	55<br>36	-	120°/120°
flexible FOS	2.2	Olympus	Rhinolaryngoscope<br>Laryngoscope	ENF-XP<br>LF-P	30<br>60	-<br>-	130°/130°<br>120°/120°
		Pentax	Nasopharyngo Laryngoscope	FNL-7RP3	30	-	130°/130°
	2.4	Pentax	Intubation scope	FI-7P/7BS	60	-	130°/130°
	2.5	Karl Storz	Laryngo-fiberscope	11101 SK<br>11101 SP	27<br>37	-<br>-	180°/90°<br>180°/90°
	2.7	Pentax	Bronchoscope	FB-8V	60	+	180°/130°
	2.8	Karl Storz	Intubation-fiberscope<br>Broncho-fiberscope<br>Broncho-fiberscope	11301 AA1<br>11003 BC<br>11005 BC	65<br>54<br>70	+<br>+<br>+	140°/140°<br>170°/120°<br>170°/120°
	2.85	Karl Storz	FIVE (Flexible Intubation Video Endoscopes) 3.0® Intubation Endoscope	11301 ABXK (ETT ID 3.0 mm)	51.5		140°/140°
	3.0	Pentax	Intubation scope	FI-9BS	60	+	130/°130°
	3.1	Olympus	Laryngoscope	LF-DP	60	+	120°/120°
	3.4–3.5	Pentax	Intubation scope<br>Bronchoscope<br>Nasopharyngo Laryngoscope	FI-10P2/10BS<br>FB-10V<br>FNL-10RP3/10RBS	60<br>60<br>30	+<br>+<br>-	130°/130°<br>180/°130°<br>130°/130°
		Karl Storz	Laryngo-fiberscope<br>Laryngo-fiberscope	11001 RD<br>11101 RP	34<br>30	+<br>-	180°/100°<br>180°/90°
	3.7	Karl Storz	Intubation-fiberscope<br>Broncho-fiberscope	11302 BD2<br>11002 BD	65<br>54	+<br>+	140°/140°<br>180°/100°
	4.1–4.2	Olympus<br>Pentax	Intubation/Laryngoscope<br>Intubation scope	LF-2/GP<br>FI-13P/13BS	60<br>60	+<br>+	120°/120°<br>160°/130°
	4.9	Pentax	Bronchoscope<br>Nasopharyngo Laryngoscope	FB-15V<br>FNL-15RP3	60<br>30	+<br>-	180°/130°<br>130°/130°
	5.0	Karl Storz	Laryngo-fiberscope<br>Broncho-fiberscope	11001 UD<br>11001 BN	23<br>54	+<br>+	180°/100°<br>180°/100°
	5.1–5.3	Olympus<br>Pentax<br>Karl Storz	Laryngoscope<br>Intubation scope<br>Intubation-fiberscope	LF-TP<br>FI-16BS<br>11301 BN1	60<br>60<br>65	+<br>+<br>+	180°/130°<br>160°/130°<br>140°/140°

OD, outer diameter; FOS, fiberoptic scope; ID, inner diameter
