# Supplementary material for: Difficult Airway Management in Neonates and Infants: Knowledge of Devices and a Device-Oriented Strategy
Source: Front Pediatr. 2021 May 7;9:654291. doi: 10.3389/fped.2021.654291 (PMC8138561; doi:10.3389/fped.2021.654291)
Supplement: Supplementary file 4 [file Data_Sheet_4.PDF]

Supplementary Table S4. The device-oriented strategy for neonate/infant difficult airway management

Solutions	Device	Commercially available products
1	Video Laryngoscopes	C-MAC® Video Laryngoscope<br>GlideScope AVL®<br>McGrath MAC®<br>AirWay Scope with PBlade®<br>AirTraq®<br>TruView PCD Pediatric®<br>King Vision aBlade®
2	Supraglottic airway device	LMA Classic®, LMA Unique®, LMA ProSeal®, LMA Supreme®<br>air-Q SP®<br>i-gel®<br>Aura-i®, AuraGain®
3	Fiberscope & Intubation endoscope	Olympus ENF-XP®, LF-P®<br>Pentax FNL-7RP3®<br>Karl Storz FIVE 3.0®, BRAMBRINK® Intubation Endoscope
